# Decoupling the Conflicting Evaluative Meanings in Automatically Activated Race-Based Associations

**DOI:** 10.1177/01461672231156029

**Published:** 2023-02-27

**Authors:** Suraiya Allidina, Elizabeth U. Long, Wyle Baoween, William A. Cunningham

**Affiliations:** 1 University of Toronto, Ontario, Canada; 2 HRx Technology, Vancouver, British Columbia, Canada

**Keywords:** implicit bias, oppression awareness, implicit measurement, prejudice

## Abstract

Implicit measures of attitudes have classically focused on the association between a social group and generalized valence, but debate exists surrounding how these associations arise and what they can tell us about beliefs and attitudes. Here, we suggest that representations of oppression, which relate positively to implicitly measured prejudice but negatively to explicitly measured prejudice, can serve to decrease the predictive validity of implicit measures through statistical suppression. We had participants complete a Black–White implicit association test (IAT) and an IAT measuring representations of oppression, and find that oppression-related representations statistically suppress the relation between IAT scores and explicit attitudes, such that accounting for these representations increases the total amount of variance explained by implicit measures. We discuss the implications of this work both for practical matters around use of the IAT and for theoretical debates on the conceptualization of valence in implicit attitudes.

Over the past 30 years, attitude measurement has increasingly relied on reaction time-based “implicit” measures to better understand attitudes toward controversial topics where people might be unwilling or unable to report their attitudes. Following from Dovidio and Fazio’s seminal work in the 1980s (Dovidio et al., 1986; Fazio et al., 1986), researchers began measuring attitudes by examining the simple association between an attitude object and abstracted concepts of positive or negative valence. These new measures demonstrated that aspects of valence might be detectable from reaction times in the milliseconds, suggesting that automatic activation of attitudes may be possible. Yet, despite the excitement about these measures, much is still unknown. In particular, debate remains around how negative associations^1^ on measures such as the IAT arise and what these associative scores can tell us about the beliefs and attitudes of the person taking the test. Here, we explore the semantic content of implicit attitudes by examining how representations linking Black people with oppression may shape the implicit measurement of racially prejudiced attitudes and impact the predictive validity of the IAT through statistical suppression.

One way that the semantic content of implicit attitudes has been explored has been to examine how specific stereotypical beliefs about social groups relate to the generalized affective prejudice measured by the test. Affective prejudice often co-occurs with more specific stereotypical beliefs, whether implicit or explicit. Kurdi and colleagues (2019) have demonstrated that implicit attitudes are closely related to implicit beliefs, and in some cases, the two are even redundant with each other. In fact, different types of beliefs and emotions can contribute to negative attitudes toward different groups. Implicitly measured prejudice toward gay people, for example, tends to reflect disgust, whereas prejudice toward Arab people instead reflects anger (Dasgupta et al., 2009; but see Dang et al., 2020). In this way, the negativity assessed by common implicit measures conflates different types of beliefs or emotions that cannot be disambiguated by only assessing associations with generalized positive or negative affect. Although the relation is likely bidirectional, these specific beliefs may drive some of the more general attitudinal negativity seen on measures such as the IAT.

Research on the semantic content of implicit attitudes has typically focused on stereotypical negative beliefs about groups that are the target of prejudice. This focus is largely justified, as many of the beliefs contributing to negative attitudes are likely related to dislike of the group and are, therefore, themselves negative. There is at least one notable exception, however, in the form of egalitarian representations linking a group with oppression. Since oppression is a negatively valenced concept, associating a group with oppression produces positive scores on the IAT (Uhlmann et al., 2006). In other words, someone with stronger representations linking a particular group (e.g., Black people in the United States) with concepts of oppression or disadvantage is more likely to have IAT responses typically indicative of anti-Black attitudes than someone without these representations. However, these same oppression-related representations are actually related to more positive explicit attitudes toward the group (Uhlmann et al., 2006), suggesting that these IAT responses may not necessarily be indicative of prejudice. Supporting this idea that positive or sympathetic beliefs can sometimes underpin negative representations, Lee and colleagues (2018) found that generic negative affect triggered by Black faces could be interpreted as either fear or empathic concern, with differing effects on racial bias. Along the same lines, Andreychik and Gill (2012) demonstrated that IAT scores linking Black people with negativity differentially predicted explicit attitudes and empathic concern depending on participants’ beliefs about the causes of negative outcomes experienced by Black people. Thus, representations related to oppression which may result from more positive or sympathetic beliefs about a group may inflate estimates of prejudiced implicit attitudes for more egalitarian-minded individuals while simultaneously producing more positive explicit attitudes. The diverging relations of oppression-related representations with implicit and explicit measures of prejudice may lead to some interesting effects when trying to predict explicit attitudes or behavioral intentions from implicit measures. In particular, these representations with opposite effects on implicit and explicit measures may decrease the ability of the IAT to predict relevant outcomes, such as behavioral manifestations of racism or commitment to racial justice, through statistical suppression. We suggest that accounting for these oppression-related representations is, therefore, necessary to adequately measure prejudiced implicit attitudes.

To the degree that associating a disadvantaged group with oppression^2^ can actually lead to positive affect toward the group, it is possible that such associations may lead to suppressor effects when using the standard IAT. Suppression is a statistical phenomenon that occurs when the addition of a third variable to the regression between X and Y increases the strength of the relation between X and Y (Conger, 1974; MacKinnon et al., 2000). Suppression analyses can be thought of in the same language as mediation (and in fact, the two are statistically equivalent; MacKinnon et al., 2000) with one important difference. In mediation, adding a third variable to the regression predicting Y from X causes the direct path between X and Y to *decrease*, as some of that variance has now been attributed to the indirect path. Suppression follows the same logic, except that adding a third variable to the regression causes the direct path between X and Y to *increase*. This is because the suppressor variable relates in opposite ways to X and Y, such that the direct and indirect paths have opposite signs (MacKinnon et al., 2000). A classic hypothetical example used to explain suppression occurs when trying to predict performance on a routine cognitive task from IQ (McFatter, 1979). In this example, the direct path between IQ and performance is positive: the higher someone’s IQ, the better they perform on the task. However, the story is complicated when we introduce a third variable, boredom, into the equation and examine the indirect path through boredom. This indirect path from IQ through boredom to task performance will be negative, since IQ positively predicts boredom and boredom in turn predicts worse performance on the task (more errors). IQ, therefore, positively predicts performance through a direct path but negatively predicts performance through an indirect path. When boredom is not explicitly accounted for in the model, these two paths with opposite signs are conflated in the single path going from IQ to performance. When boredom is explicitly entered into the model, however, this variance can be properly separated into the direct and indirect paths. In this way, entering boredom into the model causes the direct path from IQ to performance to increase in magnitude, the classical indicator of suppression.

Applying this logic to the current research suggests that representations related to oppression may statistically suppress the relation between implicit and explicit measures of prejudicial attitudes. In other words, scores on the standard IAT may relate to explicit measures through two pathways, direct and indirect, with opposite signs (Figure 1). First is the direct path that is typically assumed, in which being faster to associate Black people with negatively valenced concepts predicts more prejudiced explicit responses (orange path in Figure 1). Second is an indirect pathway in which these associations are not directly prejudiced, but instead relate to greater associations of Black people with oppression and White people with privilege (blue path in Figure 1). These associations with oppression and privilege should in turn predict less prejudiced explicit responses (black path in Figure 1), as those who have stronger representations of the systemic oppression of Black people are more likely to be sympathetic to their plight. In other words, the oppression-related associations may act as a suppressor that weakens the relation between Black–White IAT scores and explicit attitude measures. If such suppression is occurring, removing the variance associated with these oppression-related associations from IAT scores (by entering a score for oppression-related associations into the model) should provide a more “pure” assessment of implicit attitudes that correlates more strongly with explicit attitudes.

**Figure 1.**
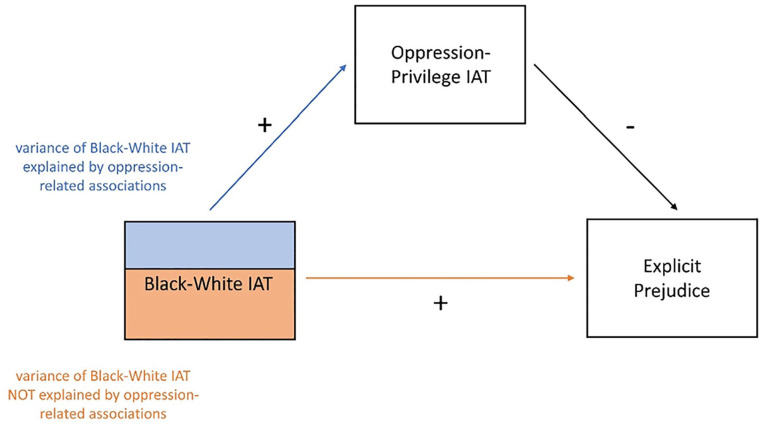
Model Depicting the Relation Between Scores on Each IAT and Explicit Attitudes. *Note.* Scores on the Black-White IAT may relate to explicit attitudes through two pathways: a direct path (orange) in which greater Black-White IAT scores predict more prejudiced explicit attitudes, and an indirect path in which greater Black-White IAT scores predict greater associations with oppression and privilege (blue), which in turn predict *less* prejudiced explicit attitudes (black). IAT = implicit association test.

Suppression of the relation between implicit and explicit measures by oppression-related representations may also help to explain a second curious effect that has been found on the IAT. This is the “right bias” posited by Blanton and colleagues (2015) who suggest that rather than having a neutral zero point, IAT scores are biased toward the right end of the measure such that even those who show no behavioral discrimination still have positive IAT scores indicative of prejudice toward Black people. The model proposed here in which oppression-related representations suppress the relation between implicit and explicit measures may help to explain any such right bias, as even those who hold no anti-Black implicit attitudes would be expected to score positively on the IAT due to knowledge of Black people’s oppression. Therefore, if we find evidence that oppression-related representations suppress the relation between IAT scores and explicit attitudes, we can examine whether controlling for these representations decreases or eliminates the right bias. Together, evidence of suppression and its resulting effects on the right bias would suggest that representations of Black people as being linked with oppression need to be disambiguated from general negative representations of Black people based on dislike, with important implications both for better measurement of implicit attitudes and for the conceptualization of valence in implicit attitudes.

In this research, we examine the challenges that oppression-related representations may pose for the predictive validity of the IAT by decomposing the variance of the Black–White IAT into variance associated with representations of oppression and variance that is independent of these representations (and that presumably more directly taps dislike or hostility). We had participants complete two sets of IATs: a classic Black–White IAT measuring associations of Black and White faces with positive and negative words, and an Oppression–Privilege IAT (Uhlmann et al., 2006) measuring associations of Black and White faces with words related to oppression and privilege. We then generated “cleansed” scores by removing the variance of the oppression-related representations from the classic IAT, using these to test for suppression and reduction in the right bias, as described above. To lend further validity to the use of explicit measures as a criterion, we aimed to collect an additional set of measures that would allow us to examine whether any suppressor effects are similarly reflected in real-world beliefs and political positions. We therefore also asked participants to rate their favorability toward a series of policies and social movements in the United States. Finally, to quantify the magnitude of these effects while controlling for method-specific variance (such as that resulting from individual differences such as executive control; Gawronski, 2019; Ito et al., 2015; Klauer et al., 2010), we had participants complete a baseline IAT in which they associate positive and negative images with positive and negative words. Overall, we aim to investigate whether accounting for oppression-related representations increases the prediction of explicit attitude measures and policy-related beliefs from implicit attitude measures, with important implications for both measurement and prejudice reduction.

## Methods

### Overview

Participants completed two sets of three IATs: a classic Black–White IAT measuring associations of Black and White faces with positive and negative words (Greenwald et al., 1998), an Oppression–Privilege IAT measuring associations of Black and White faces with words related to oppression and privilege (Uhlmann et al., 2006), and a baseline IAT measuring associations of positive and negative images with positive and negative words. They also completed a series of questionnaires assessing their explicit attitudes, including our primary outcomes of symbolic racism and blatant prejudice, as well as additional measures assessing their motivations for responding without prejudice and their feelings toward various policies and social movements. We preregistered our study design, planned sample size, and exclusion criteria at https://osf.io/phbq4/?view_only=c89b2ae608384ac494e16f0ef1497b2f.^3^ Data and analysis code for these studies can be found on the Open Science Framework (OSF) entry for this project at https://osf.io/b9s5d/?view_only=bec0c144229c498e8e9f6525f24fff66. We report all manipulations, measures, and exclusions in these studies.

### Participants

Two convenience samples of U.S. participants were recruited using TurkPrime crowdsourcing software (Litman et al., 2017) and paid US$3.00 for completing the study. In Study 1, we collected an initial exploratory data set of 300^4^ participants, of which 297 completed the main measures of the study. After applying our preregistered exclusion criteria (see below), 265 participants were left in the final sample for analyses (mean age = 37.1; 118 females, 146 males, and 1 other gender; 197 White, 17 East/Southeast Asian, 16 Black,^5^ 16 Hispanic, 16 mixed race, 2 South Asian, and 1 Indigenous). In Study 2, we collected a confirmatory data set of 750 participants, of which 744 completed the main measures of the study. After applying our preregistered exclusion criteria (see below), 626 participants were left in the final sample for analyses (mean age = 37.2; 319 females, 303 males, 3 other genders, and 1 did not specify gender; 494 White, 51 Black (Note 5), 27 East/Southeast Asian, 24 Hispanic, 18 mixed race, 5 other ethnicities, 5 South Asian, and 2 Indigenous).

We chose to collect a very large sample to maximize power within budgetary constraints. Indeed, sensitivity analyses indicate that after exclusions, we had 80% power to detect suppressor slopes as small as 0.4 in Study 1 and 0.23 in Study 2. Sensitivity analyses were conducted using the powerMediation (Qiu, 2021) package in R (R Core Team, 2021) to determine the minimal detectable slope for a mediator given our final sample sizes of 265 in Study 1 and 626 in Study 2.

### Procedure

Participants first completed a practice task to become acquainted with the IAT. They then completed the first set of three IATs in randomized order (the Black–White IAT, the Oppression–Privilege IAT, and the Positive–Negative IAT), followed by the questionnaires, and then ended with the second set of three IATs (again in randomized order).

#### Practice IAT Task

Before completing the main experimental tasks, participants completed a practice IAT in which they categorized pictures of cats and dogs (drawn from the Open Affective Standardized Image Set (OASIS) image library; Kurdi et al., 2017) and words describing fruits and vegetables. The purpose of this practice task was to acquaint participants with the mechanics of the IAT without the suggestion that the pictures and words that they were categorizing might be associated in some way. In the practice task, participants first completed eight trials with “Dog” and “Vegetable” on the right side of the screen and “Cat” and “Fruit” on the left side, after which they completed a version of the task in which the labels “Dog” and “Cat” switched places.

#### Black–White, Oppression–Privilege, and Positive–Negative IATs

Following the practice IAT, all participants completed three different versions of the IAT (Greenwald et al., 1998) in a randomized order: the standard Black–White race IAT, the Oppression–Privilege IAT developed by Uhlmann and colleagues (2006), and a Positive–Negative IAT developed for the purposes of the present study, which paired positively and negatively valenced pictures with positively and negatively valenced words. In the Black–White IAT, images of Black and White faces were associated with positively and negatively valenced words (Positive: *love, peace, honest, lucky, gift, happy, laughter, paradise*; Negative: *abuse, filth, grief, disaster, ugly, evil, rotten, agony*). The category labels presented to participants for this task were “Black” and “White” (for race) and “Positive” and “Negative” (for valence). All stimuli were taken from the Project Implicit website (Nosek et al., 2007). The Oppression–Privilege IAT used the same images used in the Black–White IAT and associated them with words relating to the categories “Oppression” (*oppressed, victimized, mistreated, brutalized*) and “Privilege” (*privileged, rulers, dominant, powerful*), previously used by Uhlmann and colleagues (2006). This IAT used the same category labels as the Black–White IAT for race (“Black” and “White”) and the same labels as in Uhlmann et al. (2006) for the oppression and privilege words (“Oppressed” and “Privileged”). Finally, to establish an “upper bound” on potential IAT scores, participants completed the Positive–Negative IAT, which paired positively and negatively valenced images drawn from the OASIS image library (Kurdi et al., 2017) with the same set of positively and negatively valenced words used in the Black–White IAT. The category labels presented to participants in this IAT were “Positive Image,” “Positive Word,” “Negative Image,” and “Negative Word.” By assessing associations between two types of explicitly negative stimuli and two types of explicitly positive stimuli, this Positive–Negative IAT serves as a measure of the maximum possible effect we would theoretically expect to see on an IAT for each subject. In other words, this IAT serves to account for “method-specific variance” (Mierke & Klauer, 2003; Teige-Mocigemba et al., 2008), or any factor that increases a participant’s scores on IATs in general but that does not relate to the specific attitude of interest. This could include factors such as executive control abilities, motor reaction time, and the size of the screen and keyboard participants used to complete the task.

Each IAT consisted of two blocks of 40 trials, one with all congruent trials and one with all incongruent trials. Due to an error, eight participants in the final data completed more than 40 trials of one of the IAT blocks. We include these participants’ responses in all analyses reported below, but excluding these participants from analysis did not affect the conclusions. No practice trials were included in these IATs, since participants completed a separate Practice IAT to learn about the task. Participants completed each IAT twice, once with the incongruent block first and once with the congruent block first, for a total of six IATs with 80 trials each. The order in which participants completed the incongruent-first and congruent-first IATs was counterbalanced across participants. To decrease boredom and fatigue with multiple IAT administrations, we had participants complete the first set of IATs, followed by the questionnaires (described below), which were then followed by the second set of IATs.

IAT scores were filtered in the following ways according to our preregistered protocol and then transformed into D-scores (Greenwald et al., 2003). We largely followed the protocols described by Greenwald and colleagues (2003), except for our treatment of incorrect trial latencies and trial outliers. As incorrect trials were repeated until the participant responded correctly, response latencies for incorrect trials were added to the latency for the eventual correct response. To correct for potential outliers on the IAT, we deleted trials if their response latencies were less than 400 ms or greater than 3,000 ms, or if the participant provided the incorrect response for that trial three times in a row or more. However, our effects hold if the scoring algorithm suggested by Greenwald and colleagues (2003) is instead used, with latencies instead filtered out if they are above 10,000 ms and latencies for incorrect trials replaced by the mean latency for correct trials plus a penalty of 600 ms.^6^ Finally, incorrect responses were deleted from analysis. For each participant and IAT, mean latencies were calculated separately for congruent trials (e.g., where White faces were paired with positive words and Black faces with negative words) and incongruent trials (e.g., where Black faces were paired with positive words and White faces with negative words). To generate scores for each participant, the congruent latencies were subtracted from the incongruent latencies, such that larger indices for the Black–White and Oppression–Privilege IAT represented stronger association of White faces with words related to positivity or privilege and Black faces with words related to negativity or oppression. For the Positive–Negative IAT, larger indices represented stronger associations of positive pictures with positive words and negative pictures with negative words. These difference scores were then divided by the pooled standard deviation calculated across congruent and incongruent blocks to get one D-score for each of the six IATs that each participant completed. Scores from the two IATs of the same type were then averaged to get three D-scores per participant: a Black–White IAT score, an Oppression–Privilege IAT score, and a Positive–Negative IAT score. Following Greenwald and colleagues (2003), participants who had reaction times faster than 300 ms on more than 10% of IAT trials were excluded (see end of Methods for final sample sizes).

To calculate the reliability of each IAT, we computed the permutation-based split-half reliability with a Spearman-Brown correction using 5,000 permutations (Parsons et al., 2019). Reliability was similar for each of the three IATs, yielding estimates of 0.82 for the Oppression–Privilege IAT, 0.83 for the Black–White IAT, and 0.83 for the Positive–Negative IAT.

### Questionnaires

Following completion of the IATs, participants completed a series of questionnaires assessing their explicit racial attitudes and political views.

#### Explicit Racial Attitudes

In accordance with our preregistration, explicit racial attitudes were measured using the Blatant Prejudice Scale (Pettigrew & Meertens, 1995) and the Symbolic Racism Scale (Henry & Sears, 2002). The Blatant Prejudice Scale consists of nine items meant to measure overt racial animosity toward Black people as a group, notably perceptions of Black people as threatening and/or inferior (e.g., “*Black Americans come from less able races and this explains why they are not as well off as most White people.*”) and a desire to avoid intimacy or contact with them (e.g., “*I would not mind if a suitably qualified Black person was appointed as my boss.*” [reverse-coded]). The original Blatant Prejudice scale consisted of 10 items; however, one item that assumed the participant was a member of the majority group was dropped, as our sample consisted of people from different races and ethnicities. The Symbolic Racism Scale consists of eight items meant to capture more subtle, “modern” racism thought to have emerged in America post-Jim Crow, which involves racial resentment (e.g., “*Over the past few years, Black people have gotten more economically than they deserve.*”), denial of existing racial oppression (e.g., “*Discrimination against Black people is no longer a problem in the United States.*”), and blame toward Black people and communities for the negative outcomes they experience as a group (e.g., “*It’s really a matter of some people not trying hard enough; if Black people would only try harder they could be just as well off as White people.*”). The language of these scales was updated for a modern, American audience. In the Blatant Prejudice Scale, originally developed in the United Kingdom, “West Indians” was changed to “Black people” or “Black Americans,” whereas “British people” was changed to “White people” or “White Americans.” In the Symbolic Racism Scale, “blacks” was changed to “Black people” and “whites” was changed to “White people.”

#### Secondary Attitudinal Measures

In addition to directly assessing explicit racial prejudice using our preregistered scales (Blatant Prejudice and Symbolic Racism), we collected other measures of attitudes and behaviors associated with racial prejudice (and lack thereof) to serve as additional means of evaluating the predictive value of the different IAT scores. We use these measures in a similar manner as the explicit attitudes scales, examining whether the relations between scores on the Black–White IAT and these measures are suppressed by scores on the Oppression–Privilege IAT.

##### Motivations to Respond Without Prejudice

Participants in Study 2 also completed the Internal Motivation to Respond Without Prejudice Scale (IMS) and the External Motivation to Respond Without Prejudice Scale (EMS; Plant & Devine, 1998). The IMS consists of five questions assessing the degree to which participants attempt to behave without prejudice toward Black people due to internal, personal motivations (e.g., “*I attempt to act in nonprejudiced ways toward Black people because it is personally important to me.*”), whereas the five questions of the EMS assess the degree to which external, social factors motivate nonprejudiced behaviors (e.g., *“I try to hide any negative thoughts about Black people to avoid negative reactions from others.”*). Participants responded to these items on a 7-point scale, ranging from “strongly disagree” to “strongly agree,” with higher scores on each scale indicating higher internal or external motivations for controlling prejudice.

##### Feeling Thermometers

In addition, participants completed “feeling thermometer” ratings of their general affect toward 28 groups, movements, and concepts that are controversial in modern-day America (Abortion rights, Affirmative action, Black Lives Matter, Combatting climate change, Europe, Feminism, Fox News, Gay marriage, Government, Green New Deal, Gun control, Hate speech laws, Immigration, Mainstream Media, Marijuana legalization, MeToo, NATO, Political correctness, Reparations for slavery, School lunch programs, The KKK, The NAACP, The police, Universal Health care, Unrestricted free speech, U.S. military, War on Terror, and Welfare). Some of these items are racially charged (e.g., “affirmative action” and “reparations for slavery”), while some are less so (e.g., “the #MeToo movement” and “combating climate change”) but nevertheless tend to be highly polarized. Items were rated from 0 (“Extremely negative”) to 100 (“Extremely positive”), and participants were given the option to check a “Not applicable” button for any items with which they lacked familiarity. Two additional items (“Human Biodiversity” and “The Intellectual Dark Web”) were rated in Study 1 but were dropped in Study 2 as a large number of participants were not familiar with them.

##### Political Attitudes

Participants also completed questionnaires assessing explicit attitudes toward conservative, egalitarian, and authoritarian ideologies, as well as rating the plausibility of various causes (historical and present oppression, differences in culture, and differences in biology) for differences in achievement between Black and White Americans. In addition, general political attitudes were measured using a 10-point scale of affiliation with the “left” versus “right” of the political spectrum. These data were collected for the purpose of validating these measures outside of the undergraduate population for use in future studies and will not be discussed further in the present article.

#### Data Exclusions

Preregistered exclusion criteria were used to remove participants who may not have been paying attention during the study. To remove those who may have been randomly responding in the questionnaires, participants whose responses to positively worded and negatively worded questionnaire items were not sufficiently aligned were excluded as follows. Each participant’s responses to the positively worded items and the reverse-coded items on the two explicit attitude scales were averaged, and then, scores on the positive items were predicted from the reverse-coded items in a linear model across both studies. The residuals of this model were then examined, and participants were excluded if the absolute value of their standardized residual was greater than 3 (indicating that their responses to positively worded and reverse-coded items were vastly different). In total, 32 participants were excluded from Study 1 (29 for fast reaction times, 1 for inconsistent questionnaires, and 2 for both), leaving a final sample size of 265. A total of 118 participants were excluded from Study 2 (97 for fast reaction times, 5 for inconsistent questionnaires, and 16 for both), leaving a final sample size of 626. Across both studies combined, this left a total of 891 participants whose data were analyzed.

## Results

With a few minor exceptions, the two studies that comprise this project were virtually identical. As noted earlier, the first data set was collected as an exploratory data set to gather initial support for our hypotheses, and the second data set was collected as confirmatory. We report the results from both studies in the text below and in all tables. Descriptive statistics for the IATs and explicit measures of prejudice are presented in Tables 1 and 2 for Studies 1 and 2, respectively.

**Table 1.** Study 1: Means, Standard Deviations, and Correlations With Confidence Intervals.

Variable	*Mean* (*SD*)	1	2	3	4	Congruent latency	Incongruent latency
1. Black–White IAT	0.28 (0.37)					866.66	947.03
2. Oppression–Privilege IAT	0.24 (0.35)	.53** [.44, .61]				898.36	971.91
3. Positive–Negative IAT	1.38 (0.49)	.28** [.16, .39]	.26** [.14, .37]			911.58	1393.67
4. Symbolic Racism	0.96 (0.73)	.25** [.13, .36]	.03 [−.09, .15]	−.00 [−.12, .12]			
5. Blatant Prejudice	1.15 (0.94)	.22** [.10, .33]	.02 [−.11, .14]	−.03 [−.15, .09]	.71** [.64, .76]		

*Note. SD* is used to represent the standard deviation. Values in square brackets indicate the 95% confidence interval for each correlation. For each IAT, we also indicate the mean latencies in milliseconds for the congruent conditions (i.e., Black/bad, White/good for the Black–White IAT; Black/oppressed, White/privileged for the Oppression–Privilege IAT; and positive picture/positive word, negative picture/negative word for the Positive–Negative IAT) and the incongruent conditions (which correspond to the opposite pairings). IAT = implicit association test.

* *p* < .05. ** *p* < .01.

**Table 2.** Study 2: Means, Standard Deviations, and Correlations With Confidence Intervals.

Variable	*Mean* (*SD*)	1	2	3	4	5	6	Congruent latency	Incongruent latency
1. Black–White IAT	0.33 (0.36)							854.20	948.82
2. Oppression–Privilege IAT	0.29 (0.35)	.29** [.22, .36]						883.92	969.03
3. Positive–Negative IAT	1.44 (0.49)	.15** [.07, .23]	.23** [.16, .31]					894.10	1390.73
4. Symbolic Racism	1.03 (0.74)	.18** [.10, .25]	−.16** [−.24, −.08]	−.04 [−.12, .04]					
5. Blatant Prejudice	1.19 (0.97)	.18** [.11, .26]	−.13** [−.21, −.06]	−.04 [−.12, .04]	.69** [.64, .73]				
6. IMS	4.78 (1.26)	−.14** [−.22, −.06]	.11** [.03, .19]	.05 [−.03, .13]	−.54** [−.59, −.48]	−.64** [−.69, −.60]			
7. EMS	2.21 (1.51)	.13** [.05, .21]	.04 [−.04, .11]	−.00 [−.08, .07]	.27** [.19, .34]	.39** [.32, .45]	−.23** [−.30, −.15]		

*Note. SD* is used to represent the standard deviation. Values in square brackets indicate the 95% confidence interval for each correlation. For each IAT, we also indicate the mean latencies in milliseconds for the congruent conditions (i.e., Black/bad, White/good for the Black–White IAT; Black/oppressed, White/privileged for the Oppression–Privilege IAT; and positive picture/positive word, negative picture/negative word for the Positive–Negative IAT) and the incongruent conditions (which correspond to the opposite pairings). IAT = implicit association test; IMS = internal motivation to respond without prejudice scale; EMS = external motivation to respond without prejudice scale.

* *p* < .05. **p < .01.

### Suppressor Effects

If oppression-related representations influence IAT scores without contributing to prejudice, these representations should suppress the relation between Black–White IAT scores and explicit attitude measures. In particular, if IAT scores predict explicit attitudes positively through a direct path but negatively through an indirect path through oppression-related representations, removing the variance associated with these representations should provide a “purer” assessment of implicit attitudes that correlates more strongly with explicitly measured prejudice (see Figure 1). In this case, controlling for the Oppression–Privilege IAT when predicting explicit attitudes from the Black–White IAT should result in a standardized coefficient that is larger than the zero-order correlation of the same two variables. As seen in Table 3, this is indeed the case: Scores on the Black–White IAT more strongly predicted both measures of explicit attitudes once scores on the Oppression–Privilege IAT were taken into account.

**Table 3.** Regression Results Using Symbolic Racism and Blatant Prejudice as the Criteria.

Predictor	beta	*t*	*r*
Symbolic racism			
Study 1			
(Intercept)			
Black–White IAT	0.33 (0.36)	4.66*** (4.53)	.25** (.25)
Oppression–Privilege IAT	−0.15 (−0.22)	−2.11* (−2.72)	.03 (−.03)
Study 2			
(Intercept)			
Black–White IAT	0.25 (0.22)	6.14*** (4.96)	.18** (.16)
Oppression–Privilege IAT	−0.23 (−0.25)	−5.80*** (−5.53)	−.16** (−.19)
Blatant prejudice			
Study 1			
(Intercept)			
Black–White IAT	0.29 (0.31)	4.17 (3.79)***	.22** (.21)
Oppression–Privilege IAT	−0.14 (−0.18)	−2.01 (−2.26)*	.01 (−.02)
Study 2			
(Intercept)			
Black–White IAT	0.24 (0.27)	6.06 (5.98)***	.18** (.21)
Oppression–Privilege IAT	−0.21 (−0.22)	−5.12 (−4.81)***	−.13** (−.14)

*Note. Beta* represents the standardized regression weights when both IATs are entered in the model, *t* represents the *t* values corresponding to these estimates, and *r* represents the zero-order correlation between each IAT and the explicit measure. A significant *t* value indicates the *beta* weight is also significant. Values in parentheses are for the subset of only White participants. Overall, *beta* values are larger than the corresponding *r* values, indicating that the relation of each IAT with the explicit measure gets stronger when controlling for variance associated with the other IAT. IAT = implicit association test.

* *p* < .05. **p < .01. ***p < .001.

**Figure 2.**
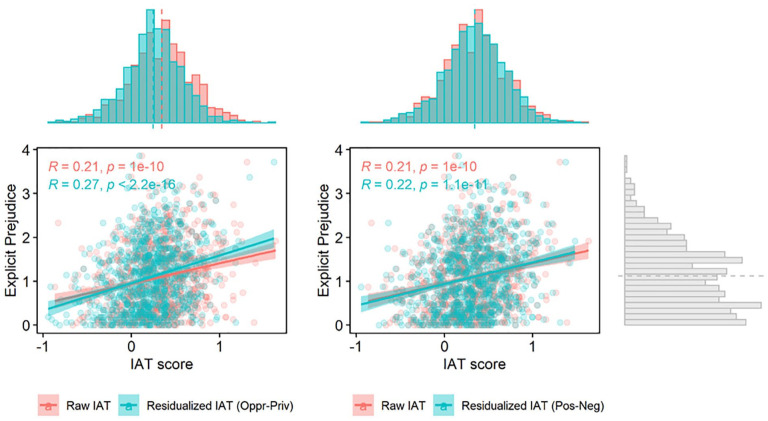
Relation of Raw and Residualized IAT Scores With Explicit Measures. *Note*. The left panel shows IAT scores residualized by removing variance associated with the Oppression-Privilege IAT. The residualized scores have an increased correlation with explicit attitudes and a leftward shift in the overall distribution of IAT scores. The right panel shows a control analysis in which we instead residualize IAT scores by scores on the Positive-Negative IAT (see *Ruling out alternative explanations* section). As expected, the same effects are not found here, suggesting that the results are not simply due to method variance shared among the IATs.

**Figure 3.**
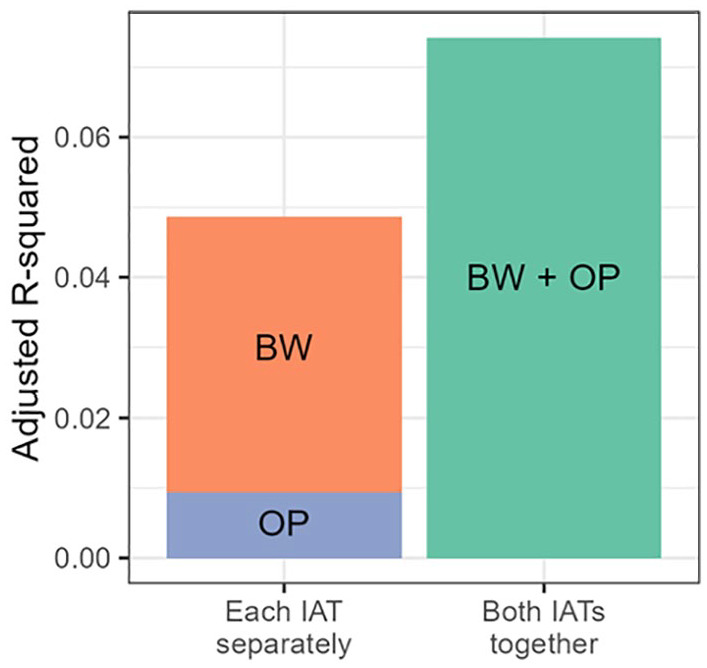
Adjusted R-Squared for Models Predicting Symbolic Racism Scores From IAT Scores. *Note*. The left bar shows the adjusted R-squared from the models predicting explicit attitudes from each IAT individually (BW = Black-White, OP = Oppression-Privilege). The height of the bar shows the summed adjusted R-squared for each of the two models combined, and the colours represent the variance explained by each IAT individually. The right bar shows the adjusted R-squared for the model predicting explicit attitudes from the two IATs combined. Due to statistical suppression, including both IATs in the model leads to more total variance explained than including each IAT in a separate model.

As a more formal test of this idea, we conducted a series of suppression analyses predicting explicit attitudes from both the Black–White IAT and the Oppression–Privilege IAT, testing for a potential indirect effect of the Black–White IAT through the Oppression–Privilege IAT. Evidence for suppression would be found in obtaining both a positive direct effect of the Black–White IAT on explicit attitudes and a negative indirect effect through the Oppression–Privilege IAT (see Figure 1). In other words, the test for suppression uses the same basic logic of mediation, except that (a) the direct path and the indirect are of different signs, and (b) the effect of the predictor variable increases rather than decreases when controlling for the suppressor variable (MacKinnon et al., 2000). All models were run using 2,000 bootstraps in the mediation toolbox in *R* (Tingley et al., 2014). As can be seen in Table 4 and visualized in Figure 2, evidence of suppression was found for both measures of explicit attitudes.^7^ As a result of this suppression, including the Oppression–Privilege IAT as a simultaneous predictor when regressing the Black–White IAT on explicit attitudes increased the total amount of variance explained above and beyond the variance explained by each IAT individually (see Figure 3). These results replicate when fit as structural equation models (see Supplemental Materials).

**Table 4.** Mediation/Suppression Results Using Explicit Attitude Scales as the Criterion.

Variable	Indirect effect	Direct effect	Total effect	Prop mediated
Study1				
Symbolic racism	−0.157 [−0.301, −0.014]*	0.645 [0.378, 0.918]***	0.488 [0.266, 0.722]***	−0.319 [−0.81, −0.026]*
Blatant prejudice	−0.191 [−0.385, −0.001]*	0.747 [0.391, 1.087]***	0.556 [0.252, 0.856]***	−0.344 [−1.063, −0.002]*
Study2				
Symbolic racism	−0.14 [−0.203, −0.085]***	0.502 [0.347, 0.668]***	0.363 [0.214, 0.517]***	−0.38 [−0.793, −0.203]***
Blatant prejudice	−0.165 [−0.247, −0.096]***	0.659 [0.446, 0.884]***	0.494 [0.292, 0.714]***	−0.328 [−0.711, −0.172]***

*Note.* The indirect effect represents the effect of the Black–White IAT on explicit attitudes through the Oppression–Privilege IAT. The direct effect represents the unique effect of the Black–White IAT on explicit attitudes after removing variance associated with the Oppression–Privilege IAT. The total effect represents the overall effect of the Black–White IAT on explicit attitudes including the direct effect and the indirect effect through the Oppression–Privilege IAT. Prop mediated describes the proportion of the total effect that goes through the mediator. A negative prop mediated represents a suppressor effect. IAT = implicit association test.

* *p* < .05. ** *p* < .01. *** *p* < .001.

### Recalibrating the “Right Bias”

Blanton and colleagues (2015) proposed that the IAT has a “right bias,” wherein individuals who show no behavioral indicators of prejudice nevertheless present as prejudiced on the IAT. While there is debate around its existence and interpretation (Cvencek et al., 2012), we examine whether such a right bias may be at least partially explained by contamination of the Black–White IAT with representations of oppression and privilege. If oppression-related representations falsely inflate estimates of prejudiced implicit attitudes, even individuals who hold no anti-Black implicit attitudes may demonstrate a “right bias” through having positive scores on the IAT due to their knowledge of the oppression faced by Black people, a potential consequence of the same suppression effects discussed above. Since we found evidence that these suppression effects are present, we conducted an additional series of analyses predicting IAT scores from explicit attitudes, examining how removing the variance of oppression-related representations influences the zero point on the Black–White IAT.

For each regression, we coded the intercept to represent the value on the Black–White IAT when the lowest score possible was given for an explicit attitude measure (i.e., the expected Black–White IAT score for a participant with no explicit bias). In a second set of regressions, we also included the Oppression–Privilege IAT as a predictor centered around a score of zero. For these analyses, the intercept represents the expected Black–White IAT score for a participant that reports no explicit bias and also does not have any association between Black/White faces and concepts of oppression/privilege. A reduction in the intercept for this model would support the idea that oppression-related representations contribute to the right bias on the IAT. When the Oppression–Privilege IAT was not considered, a significant and large positive intercept was found in both Study 1, *b* = 0.15, 95% CIs = [0.08, 0.23], *p* < .001, and Study 2, *b* = 0.22, 95% CIs = [0.18, 0.27], *p* < .001, suggesting participants who reported no explicit bias still showed a strong Black–White IAT effect (paralleling the right bias found in previous research; Blanton et al., 2015). When controlling for scores on the Oppression–Privilege IAT, however, the intercept values were considerably diminished in Study 1, *b* = 0.03, 95% CIs = [−0.04, 0.10], *p* = .392, and in Study 2, *b* = 0.10, 95% CIs = [0.04, 0.15], *p* < .001, indicating that automatic egalitarian representations contributed in part to the right bias on the Black–White IAT. The nonoverlapping confidence intervals of the intercept before and after residualizing the scores in Study 2 (though slightly overlapping in the less well-powered Study 1) indicate a significant increase in the relation between the Black–White IAT and explicit attitudes when accounting for the Oppression–Privilege IAT. Specifically, controlling for representations of oppression and privilege decreased the value of the intercept by about half in Study 2 (and even further in Study 1, though with wider confidence intervals due to the smaller sample size), indicating that approximately half of the IAT’s right bias is explained by representations of privilege and oppression. Thus, to the degree that the right bias poses a measurement problem for the IAT, considering the meaning and source of valenced representations can help to alleviate such a bias. It is important to note, however, that the intercept remains significantly different from zero even after controlling for the egalitarian representations, suggesting that factors other than awareness of societal oppression and privilege additionally influence IAT scores without influencing measures of explicit attitudes.

### Predicting Secondary Attitudinal Variables

As an additional test of our hypothesis, we repeated our analysis of suppressor effects on a number of other variables that are associated with prejudice. Similarly to the analyses of explicit attitudes, evidence for suppression here would provide further support that representations of oppression are contaminating IAT scores without actually contributing to prejudice. When predicting Motivations to Respond without Prejudice (which was only collected in Study 2), evidence for suppression was found on the IMS (indirect effect: 0.172 [0.083, 0.28], direct effect: −0.663 [−0.944, −0.383], total effect: −0.49 [−0.761, −0.224], and prop mediated: −0.344 [−0.923, −0.151]) but not the EMS (indirect effect: −0.004 [−0.11, 0.1], direct effect: 0.559 [0.208, 0.915], total effect: 0.555 [0.225, 0.893], and prop mediated: −0.005 [−0.249, 0.226]). This divergence between the IMS and EMS may reflect the greater role of oppression-related representations in driving internal motivations to be non-prejudiced, with less relevance for externally motivated behavior that is driven by social desirability.

We also repeated our analysis of suppressor effects on participants’ feeling thermometer ratings of various politically polarizing groups, policies, and movements within the United States, some of which were directly related to race (e.g., Black Lives Matter) and others which were less so (e.g., gay marriage). Overall, evidence for suppression was found for 20 out of 28 feeling thermometers (see Table 5). Examining the effects in Table 5 suggests that suppression was generally more likely to be found for items that were more directly related to race, although this is not a perfect mapping. Nevertheless, this provides further evidence that oppression-related representations artificially inflate scores on the IAT such that accounting for these representations increases the correlation between IAT scores and explicitly measured attitudes.

**Table 5.** Mediation/Suppression Results Using Feeling Thermometers as the criterion (Studies 1 and 2).

Feeling thermometer item	Indirect effect	Direct effect	Total effect	Prop mediated
**Universal health care**	4.748 [2.432, 7.166]***	−**11.019 [**−**17.39**, −4.842]***	−**6.271 [**−**12.08**, −0.552]*	−**0.742 [**−**4.628**, −0.269]*
**Gay marriage**	4.026 [1.53, 6.497]***	−**9.99 [**−**16.162**, −3.834]***	−**5.964 [**−**11.703**, −0.323]*	−**0.646 [**−**3.612**, −0.12]*
**Combatting climate change**	3.297 [1.172, 5.347]**	−**8.724 [**−**13.829**, −3.554]**	−**5.427 [**−**10.371**, −0.478]*	−**0.59 [**−**3.575**, −0.119]*
**Hate speech laws**	3.281 [0.975, 5.777]**	−**9.272 [**−**15.338**, −2.911]**	−**5.991 [**−**11.827**, −0.407]*	−**0.545 [**−**3.784**, −0.087]*
**The KKK**	−**1.92 [**−**3.096**, −0.807]**	5.673 [2.617, 8.641]***	3.753 [0.888, 6.554]**	−**0.507 [**−**2.167**, −0.174]**
War on Terror	−2.672 [−4.897, −0.636]**	8.248 [2.226, 13.677]**	5.576 [0.148, 10.578]*	−0.467 [−2.804, 0.1]
**Green New Deal**	4.298 [1.727, 7.168]***	−**14.076 [**−**20.822**, −7.593]***	−**9.778 [**−**16.179**, −3.668]***	−**0.429 [**−**1.304**, −0.162]**
**Immigration**	4.473 [2.451, 6.801]***	−**15.551 [**−**20.756**, −10.27]***	−**11.079 [**−**15.977**, −6.124]***	−**0.402 [**−**0.852**, −0.197]***
**Welfare**	4.184 [2.072, 6.182]***	−**14.542 [**−**19.916**, −9.412]***	−**10.359 [**−**15.242**, −5.579]***	−**0.401 [**−**0.855**, −0.18]***
**Affirmative action**	4.504 [2.305, 6.88]***	−**17.155 [**−**23.146**, −11.231]***	−**12.651 [**−**18.112**, −7.157]***	−**0.353 [**−**0.769**, −0.161]***
Mainstream Media	1.737 [−0.188, 3.656]	−6.28 [−11.525, −1.202]*	−4.543 [−9.257, 0.4]	−0.352 [−2.64, 0.964]
Marijuana legalization	1.773 [−0.306, 3.961]	−6.471 [−12.344, −0.684]*	−4.698 [−10.262, 0.777]	−0.342 [−3.306, 1.559]
**Black Lives Matter**	5.193 [2.854, 7.762]***	−**20.309 [**−**26.465**, −13.787]***	−**15.116 [**−**20.877**, −8.826]***	−**0.338 [**−**0.69**, −0.166]***
**School lunch programs**	2.479 [0.808, 4.216]**	−**9.946 [**−**14.067**, −5.574]***	−**7.466 [**−**11.461**, −3.348]***	−**0.325 [**−**0.903**, −0.102]**
**Abortion rights**	2.922 [0.457, 5.335]*	−**12.293 [**−**18.72**, −5.695]***	−**9.371 [**−**15.313**, −3.3]**	−**0.311 [**−**0.956**, −0.046]*
**Gun control**	3.769 [1.381, 6.365]**	−**15.866 [**−**22.576**, −9.602]***	−**12.097 [**−**18.204**, −6.152]***	−**0.31 [**−0.775, −0.1]**
**Fox News**	−3.341 [−5.535, −1.235]***	14.342 [8.559, 20.042]***	11 [5.652, 16.107]***	−0.303 [−0.702, −0.105]***
**Feminism**	3.838 [1.521, 6.115]**	−17.75 [−23.962, −11.584]***	−13.912 [−19.51, −8.037]***	−0.275 [−0.593, −0.103]**
**The NAACP**	3.023 [1.009, 5.129]**	−14.276 [−19.894, −8.644]***	−11.253 [−16.377, −5.925]***	−0.269 [−0.596, −0.083]**
**Political correctness**	2.27 [0.202, 4.435]*	−11.806 [−17.329, −6.149]***	−9.536 [−14.598, −4.525]***	−0.234 [−0.66, −0.021]*
**U.S. military**	−2.143 [−4.292, −0.006]*	11.387 [5.674, 17.1]***	9.245 [3.875, 14.579]***	−0.229 [−0.697, −0.001]*
**MeToo**	3.141 [0.963, 5.401]**	−17.511 [−23.361, −11.484]***	−14.369 [−19.967, −8.735]***	−0.218 [−0.442, −0.062]**
**Reparations for slavery**	4.391 [2.076, 6.82]***	−25.397 [−31.731, −19.122]***	−21.006 [−26.551, −15.282]***	−0.207 [−0.366, −0.098]***
The police	−0.662 [−2.654, 1.285]	13.028 [7.886, 18.471]***	12.367 [7.283, 17.509]***	−0.053 [−0.263, 0.113]
Unrestricted free speech	−0.005 [−1.859, 1.844]	1.995 [−2.987, 7.004]	1.99 [−2.709, 6.537]	0.001 [−3.615, 3.058]
Government	−0.017 [−1.77, 1.779]	1.279 [−3.561, 6.28]	1.262 [−3.172, 5.88]	0.004 [−4.492, 4.357]
NATO	2.598 [0.821, 4.597]**	−2.319 [−7.231, 2.442]	0.279 [−4.374, 4.556]	0.545 [−17.898, 18.167]
Europe	2.369 [0.913, 3.963]***	−0.314 [−4.244, 3.563]	2.054 [−1.547, 5.716]	0.833 [−10.201, 9.867]

*Note.* The indirect effect represents the effect of the Black–White IAT on feeling thermometers through the Oppression–Privilege IAT. The direct effect represents the unique effect of the Black–White IAT on feeling thermometers after removing variance associated with the Oppression–Privilege IAT. The total effect represents the overall effect of the Race IAT on feeling thermometers including the direct effect and the indirect effect through the Oppression IAT. Prop mediated describes the proportion of the total effect that goes through the mediator. A negative prop mediated represents a suppressor effect (significant effects bolded). IAT = implicit association test; KKK = Ku Klux Klan; NAACP = National Association for the Advancement of Colored People; NATO = North Atlantic Treaty Organization.

* *p* < .05. **p < .01. ***p < .001.

### Ruling Out Alternative Explanations

So far, our results suggest that representations related to oppression seem to contribute to scores on the classic Black–White IAT while having the opposite relation to explicit measures of prejudice. A potential alternative explanation is that rather than reflecting a theoretically meaningful relationship between oppression-related representations and IAT performance, our analyses have instead picked up on individual differences in general response-congruency task performance (or “method-specific variance”; Mierke & Klauer, 2003; Teige-Mocigemba et al., 2008).

To account for this possibility, we re-ran the suppression analyses with the Positive–Negative IAT in place of the Oppression–Privilege IAT. If our results are theoretically meaningful and specific to oppression-related representations, accounting for scores on the Positive–Negative IAT should not produce the same effects, as this IAT is not expected to suppress the relation between valenced representations and explicit attitudes. Indeed, including the Positive–Negative IAT did not improve the Black-White IAT’s relation with explicit measures in any analysis. Unlike for the Oppression–Privilege IAT, scores on the Positive–Negative IAT did not seem to change the relation between the Black–White IAT and symbolic racism in either Study 1 (indirect effect: *b* = −0.042; prop mediated: *b* = −0.082, *p* = .22) or Study 2 (indirect effect: *b* = −0.02; prop mediated: *b* = −0.051, *p* = .089). Similarly, no evidence for suppression by the Positive–Negative IAT was found on blatant prejudice in Study 1 (indirect effect: *b* = −0.073; prop mediated: *b* = −0.126, *p* = .091) or Study 2 (indirect effect: *b* = −0.027; prop mediated: *b* = −0.051, *p* = .11). This provides a robustness check for our analyses, suggesting that our results are not simply due to shared method variance between the IATs (see Figure 2, right panel).

As an additional test to ensure the sensitivity of our analyses investigating the right bias, we also examined whether controlling for scores on the unrelated Positive–Negative IAT would reduce the expected score on the Black–White IAT for a person with no explicit bias. We expect no effect here, as individual differences in reaction times and executive control should not influence expected scores on prejudice. Indeed, including scores on the Positive–Negative IAT, centered at its mean, did not reduce the intercept of the regression model either in Study 1 (without control: *b* = 0.15, 95% CI = [0.18, 0.27], *p* < .001; with control: *b* = 0.15, 95% CI = [0.08, 0.22], *p* < .001) or in Study 2 (without control: *b* = 0.225, 95% CI = [0.18, 0.27], *p* < .001; with control: *b* = 0.221, 95% CI = [0.17, 0.27], *p* < .001), suggesting that, as with the suppression analyses, our results on the right bias cannot simply be accounted for the shared method variance between the two IATs (see Figure 2, right panel).

Finally, an additional concern involves potential order effects on the suppression results. Specifically, the order in which participants completed the Black–White IAT and the Oppression–Privilege IAT in the first set of IATs was randomized, such that about half the participants completed each IAT first. It is possible that for those who completed the Oppression–Privilege IAT before the Black–White IAT, the concepts of oppression and privilege were made salient before completion of the standard Black–White IAT, such that participants may have interpreted the valenced words on the standard IAT as having to do with oppression and privilege rather than general negativity and positivity. Thus, the suppression results could be a result of this “recoding” of the valenced words on the standard IAT. If this recoding was responsible for our results, we would expect that the suppression would only hold for those participants who completed the Oppression–Privilege IAT first. Looking only at the first set of IATs in the exploratory first study, we find that these results are significant for those who completed the Oppression–Privilege IAT first (indirect effect: *b* = −0.221; prop mediated: *b* = −0.514, *p* = .029) but not those who completed the Black–White IAT first (indirect effect: *b* = −0.034; prop mediated: *b* = −0.068, *p* = .422). However, examining our more well-powered confirmatory Study 2 indicates that these results hold both for those who completed the Oppression–Privilege IAT first (indirect effect: *b* = −0.082; prop mediated: *b* = −0.207, *p* = .01) and for those who completed the Black–White IAT first (indirect effect: *b* = −0.031; prop mediated: *b* = −0.157, *p* = .027). Interestingly, the effect was larger for those who completed the Oppression–Privilege IAT first, suggesting that such recoding may indeed have been occurring but that it cannot fully explain our results.

Finally, to ensure that our results were not shaped by practice effects on the IATs, we conducted each of these analyses an additional two times: once with only the first set of IATs and once with only the second set of IATs. Results held across both re-analyses, suggesting that practice effects are not responsible for the findings.

## Discussion

Over the past few decades, the IAT has emerged as an immensely popular way to assess prejudice, dominating research on implicit attitudes and public discussion of implicit bias. In demonstrating that it is possible to measure attitudes from reaction times in mere milliseconds, the IAT has become a useful tool in domains where people might be unwilling or unable to report their attitudes explicitly (Kurdi et al., 2021). However, the nature of these measures, including what precisely they are measuring and their ability to predict relevant outcomes, has come under intense scrutiny and is still the subject of great debate (Blanton et al., 2006; Nosek & Sriram, 2007; Schimmack, 2021). Many have argued that despite the hype, theoretical uncertainty and poor measurement validity render the IAT unable to live up to its promise as a way to assess attitudes. Here, we suggest that some of these limitations stem from the fact that the IAT has traditionally been used to examine only one type of representations at a time (typically associations of a social group with generalized notions of positivity or negativity). By limiting its focus to one general representation at a time, the IAT has been unable to adequately capture the totality of attitudinal representations that may give rise to emergent behavior. A better understanding of the full set of representations people have may therefore improve the IAT’s ability to predict relevant outcomes, as well as help us to learn more about the nature of the constructs assessed by the IAT.

In this study, we aimed to expand the space of representations under consideration by examining representations related to oppression in addition to those related to the general valence concepts traditionally assessed by the IAT. By having participants complete a separate IAT measuring representations linking Black and White people with oppression and privilege, we demonstrated that representations related to oppression statistically suppress the relation between implicit and explicit measures of prejudice. Specifically, while associations of Black faces with oppression-related concepts in this study correlated with generally negative associations with Black faces on the standard Black–White IAT, the two made opposite predictions about explicit endorsement of prejudice and support for race-related policies and movements. Removing the variance associated with oppression-related representations therefore allowed for better prediction of both explicitly measured prejudice and support for real-world policies and movements. Critically, including both IATs simultaneously allowed us to explain more total variance than would examining each IAT individually and simply summing their explained variance (see Figure 3). Thus, we have demonstrated that expanding the space of representations assessed by the measure can lead to a better mapping of implicit measures onto both explicit attitudes and behaviorally relevant beliefs, such as support for Black Lives Matter and affirmative action policies. While future work should examine the potential suppressing effects of oppression-related representations on individual behaviors, we believe that support for policies and movements involved in advancing racial justice can provide a parallel but equally important indicator of the real-world impacts of prejudice on societal outcomes.

Importantly, statistical suppression can occur whenever there are two primary variables of interest (e.g., the Black–White IAT and explicit attitudes in the present study) that correlate positively but each relate in opposite directions to a third variable (e.g., Oppression–Privilege IAT). In other words, as long as the Oppression–Privilege IAT continues to positively predict the Black–White IAT, suppression may be found for any outcome (whether self-report or behavioral) that is negatively related to oppression-related IAT scores but positively related to Black–White IAT scores. As a result, unless oppression-related representations are taken into consideration, the Black–White IAT will always underperform in predicting that outcome. This can occur regardless of the theoretical model contributing to these relationships, such that simple consideration of how groups are associated with the general valenced concepts of “good” and “bad” will be insufficient for understanding the IAT and consideration of the wider space of representations is required.

In our examination of suppression, we have focused on how scores on the Oppression–Privilege IAT may shape the interpretation of scores on the Black–White IAT. However, because suppression can occur any time the correlational structure found here is mimicked, scores on the Oppression–Privilege IAT may themselves need to be interpreted in the context of other representations. As such, one could flip the Black–White and Oppression–Privilege IATs in the model to examine how the Black–White IAT may suppress the relation between oppression-related representations and explicit attitudes (see Supplemental Materials). Just as a high score on the Black–White IAT considered alone may be indicative of strong prejudice for one person but strong representations of oppression for another person, a high score on the Oppression–Privilege IAT could similarly relate to more negative attitudes for some people but more positive attitudes for other people, depending on the other representations or beliefs these people hold about the target group and the nature of oppression. For example, someone with strong system justification tendencies who believes that bad things happen to bad people (Jost et al., 2004; Lerner & Miller, 1978) may be more likely to have negative attitudes toward groups that are oppressed than someone without these beliefs. Thus, even the meaning of oppression-related representations may be shaped by the valence representations reflected by the Black–White IAT, furthering the idea that any given score needs to be interpreted in the context of a fuller set of representations.

Examining differences among subgroups of our participants provides further support for the importance of considering the contextual effects of other semantic concepts. Although the suppression effects hold for both groups of participants, we found that the effects were larger for participants who completed the Oppression–Privilege IAT before the Black–White IAT than for those who completed them in the reverse order. For these participants, the concepts of oppression and privilege were made salient before they completed the standard Black–White IAT, such that they may have interpreted the valenced concepts on the Black–White IAT in terms related to oppression and privilege rather than to general negativity and positivity. Although this cannot explain our effects, since significant suppression was found even for participants who completed the Black–White IAT first, it does suggest an additional source of context sensitivity. This difference in effect size further supports the idea that the meaning of negative scores is not uniform but changes depending on contextual effects like the relative salience of oppression and privilege. In fact, many contexts in which someone would complete the IAT (such as through a diversity training program or by visiting the Project Implicit website) are contexts in which oppression and privilege are very salient concepts. Thus, the participants who completed the standard IAT after the Oppression–Privilege IAT may in fact be more, not less, similar to the majority of people who are taking the IAT. This likely salience of oppression-related concepts for those taking the IAT further highlights the need to consider how these representations influence scores on the standard Black–White IAT.

### Practical Implications

In demonstrating that accounting for oppression-related representations increases the predictive validity of the IAT, this work has implications for contentious practical concerns about the use of the IAT as a measure of prejudice. Despite its popularity, critics have long argued that the test does not adequately isolate the construct of prejudice. Instead, its scores may be influenced by other negatively valenced representations, such as those stemming from empathy for suffering (Arkes & Tetlock, 2004), which do not necessarily translate into prejudicial beliefs or behavior. Specifically, because common associative measures of implicit bias do not assess information about how concepts relate (De Houwer, 2014; De Houwer et al., 2015), it is possible that at least two different types of representations could give rise to apparent negative evaluations of Black people on associative measures such as the IAT: the typically assumed hostile beliefs that Black people are negative, or more sympathetic beliefs that Black people are treated negatively in society (Arkes & Tetlock, 2004; Nosek & Hansen, 2008). Some have argued that these latter representations of a group as oppressed actually do contribute to prejudice (Uhlmann et al., 2006), perhaps as a result of the widespread belief that the world is just, such that bad things only happen to bad people (Lerner & Miller, 1978), or from the tendency for a single negative attribute to create a “halo effect,” causing other attributes to be judged negatively as well (Greenwald & Banaji, 1995; Nisbett & Wilson, 1977). However, it is possible that oppression-related representations of a group do not actually contribute to prejudice but instead simply contaminate its measurement. In other words, the knowledge that Black people are oppressed may create a negative representation (Black people are associated with the negative concept of “oppression”) without any negative (implicit or explicit) attitudes toward the group being present. Here, we have suggested that the conflation of these two types of representations poses a measurement problem for the IAT, such that sympathetic associations with the negative concept of oppression may be erroneously interpreted as prejudice by researchers using the test. However, we find that statistically controlling for these representations can improve the ability of the IAT to predict both explicit attitudes and relevant political attitudes, such as endorsement of policies and social movements related to racial justice.

The question of whether oppression-related representations contribute to prejudice or simply contaminate its measurement also has implications for efforts aimed at prejudice reduction. As racism and other forms of prejudice occupy an increasingly central place in public discussion, the potential role of oppression awareness in prejudice reduction has come under scrutiny both among the general public and in academic research on prejudicial attitudes. One side of this debate argues that the knowledge that a group is disadvantaged or oppressed can work to decrease prejudice by raising awareness of the systemic barriers faced by members of the group. This idea is seen in the widespread calls for increased education and training in the face of racism and other forms of prejudice, with the hope that a better understanding of historical and current power asymmetries will serve to decrease prejudice. The other side instead suggests that such knowledge can actually make people think of the group more negatively, perhaps in a form of “blaming the victim” or justifying the group’s bad treatment (Lerner & Miller, 1978). This perspective may be seen in responses to prejudice that aim to increase positive representations without addressing oppression directly, such as by highlighting the accomplishments of select Black individuals without addressing the current and historical oppression that excludes other Black people from similar paths. If it is the case that oppression-related representations contaminate the measurement of prejudice without actually increasing prejudicial attitudes, educating people about the systemic oppression faced by members of disadvantaged groups is unlikely to increase negative attitudes and may, therefore, be a fruitful avenue of prejudice reduction. As the case of oppression-related representations demonstrates, it is only by more fully understanding the larger set of representations that give rise to prejudiced attitudes and behaviors that we can begin to try to reduce them.

### Theoretical Implications

In addition to highlighting the necessity of more fully considering the set of representations contributing to attitudes, our findings may speak to a larger debate on the nature of implicit attitudes. Two conflicting views on the nature of associative attitudes have emerged over the past two decades, with one perspective positing that any association with a negatively valenced concept leads to negative attitudes and the other instead arguing that the specific semantic content of the representation shapes the resulting attitude. Early conceptualizations of implicit bias treat attitudes as evaluative responses distinct from the more semantic content of stereotypes (Amodio & Devine, 2006; Gilbert et al., 2012; Greenwald & Banaji, 1995). Balanced Identity Theory (Cvencek et al., 2012; Greenwald et al., 2002), for example, conceptualizes attitudes as links in an associative structure connecting social groups to valence, with connections to the “bad” node producing negative attitudes regardless of the specific content of the beliefs underlying this negative response.

In contrast, perspectives in which automatic evaluations arise from the activated semantic meaning of information in a particular context tend to prioritize the specific beliefs underlying the attitude (which may be diverse in content while having the same overall valence). Under these perspectives, the representations that produce automatic evaluations are not monolithic valenced constructs and instead need to be understood within the network of semantic associations (Cunningham & Zelazo, 2007) and contextual goals (Moors & De Houwer, 2001; Moors et al., 2005) in which they reside. For example, the nature of an evaluative response can differ depending on the target group, with implicitly measured prejudice toward gay people reflecting disgust but prejudice toward Arab people reflecting anger (Dasgupta et al., 2009 but see Dang et al., 2020). Along these lines, Neuberg and colleagues (2020) suggest that the evaluation of a group will depend on the specific threats or opportunities they afford, rather than on any simple valenced association. For example, the stereotype that Mexican immigrants provide cheap labor may lead to positive evaluations for those looking to hire such workers but negative evaluations for those competing for the same jobs (Neuberg et al., 2020).

Here, we examined a case in which negatively valenced representations (related to oppression) do not stem from negative beliefs about a group, such that they share valence but not semantic content with prejudiced representations. In finding that the specific meaning underlying the negativity (i.e., whether it arises from knowledge of oppression or not) shapes the relation of the measured representations with relevant outcomes, our results may provide support for the latter view, highlighting the importance of semantic representations and contexts rather than dichotomous concepts of valence. This is even further supported by the additional finding that those who completed the Oppression–Privilege IAT first had larger suppression effects than those who completed the Black–White IAT first (as discussed above). In other words, the immediate context of salient concepts may have shaped the way people interpreted the stimuli on the Black–White IAT. These results are also in line with accounts of attitudes that highlight the role of relational information in understanding implicit evaluation (e.g., De Houwer, 2014; De Houwer et al., 2021), suggesting that the link between “Black” and “bad” needs to be understood in light of the different possible relations between these concepts: “Black people are bad” versus “Black people are treated badly.” While future work is needed to differentiate semantic-based measures like the IAT from prejudice more generally, our findings provide initial evidence for the importance of semantic considerations in understanding implicitly measured attitudes.

Even under perspectives that prioritize semantic considerations, however, the connection between oppression awareness and prejudice reduction is unlikely to be so simple. Instead, a better understanding of how oppression-related representations interface with more reflective processes is also needed. In addition to shaping the initial activation of representations, higher-level beliefs may shape the way the representations are used in subsequent processing, potentially producing different behaviors. For example, two members of a privileged group may both associate a disadvantaged group with oppression but have different emotional responses, resulting in different behaviors. Guilt over their advantaged status may cause the first individual to distance themself from the disadvantaged group, whereas the second may successfully channel their guilt in productive ways that challenge the status quo. As similarly valenced representations can thus have diverging behavioral consequences, reducing prejudiced behaviors will require a better understanding of how these representations interact with more reflective processes such as rationalization and system justification (Jost et al., 2004).

Although we have focused primarily on how different semantic representations can give rise to different evaluative responses, other more complicated relations may be present that influence the relation between IAT measures and self-report measures. For example, as discussed above, oppression-related representations may themselves have different effects, with some of these representations contributing to aspects of negative behavior (as suggested by Uhlmann and colleagues, 2006), while other representations may act as a “gatekeeper” that prevents this negativity from being expressed explicitly (and in fact produces more positive explicit attitudes). With this in mind, an understanding of the wider space of representations, including the influence of relatively automatic egalitarianism, is required to disentangle these effects to better understand how semantic representations play into evaluative responses.

### Future Directions

As noted earlier, the pattern of suppression found here can emerge whenever measures have a similar correlational structure to those used in this research and should, therefore, not be limited to the IAT. As the implicit measure with the generally highest reliability (Bar-Anan & Nosek, 2014; Cunningham et al., 2001), the IAT was useful for our purposes in attempting to identify subtle suppressor effects. Furthermore, since the IAT is by far the most widely used of the implicit measures (and in fact is virtually synonymous with “implicit bias” both among laypeople and in some research), we chose the IAT as the best representative of implicit cognition research more broadly. However, if high enough reliability (i.e., reliability that is sufficiently high to allow for discoverable correlations between implicit and explicit measures) can be established in other measures to gain sufficient power for identifying suppressor effects, future research should attempt to replicate this work with other implicit measures. Conceptually, we expect that this finding may not be limited to the IAT but extend to other measures that are designed to tap into associations between social groups and valence (though not to measures that explicitly take into account relational information such as the relational responding task; De Houwer et al., 2015). While other work has examined how explicitly measured egalitarian beliefs relate to implicit attitudes, measuring oppression-related representations implicitly allows them to be assessed on the same level of analysis as the general negative representations that are often taken as indicators of prejudice. Other implicit tasks may be amenable to this kind of implicit measurement of oppression-related representations too; thus, future research should examine the role of oppression-related representations using other implicit measures of attitudes such as evaluative priming (Fazio, 2001) or other variants of the IAT.^8^

In addition to other kinds of implicit tasks, future research should also vary the specific stimuli used in the IAT to examine the generalizability of the findings. In this work, we used the standard stimuli taken from the Project Implicit website and used in the majority of research published on the IAT. From a conceptual point of view, one avenue for future research is to narrow or broaden the semantic space considered in the IAT stimuli to examine how this influences results. For example, if limited only to words that directly convey dislike or hatred rather than general negativity, the overlap between variance in responding on the standard Black–White IAT and the Oppression–Privilege IAT may be reduced. In addition, incorporating IATs that tap into specific implicit stereotypes (Kurdi et al., 2019) may help to further broaden the semantic space under consideration and gain a better understanding of how valenced representations on the standard IAT should be interpreted. This can provide both interesting conceptual avenues and practical considerations that can help to “purify” the measurement of implicit attitudes on the IAT.

It will also be important for future research to assess the role of suppression when examining different types of outcomes. The explicit self-report measures used here have a high degree of directness and controllability, as participants were given ample time to consider their responses. It is possible that attitude measures that share more features with implicit responding, such as a lower degree of controllability, may not be subject to such suppression effects in the same way. Future work should therefore assess the potential role of oppression-related representations in suppressing responses on less controllable measures such as speeded self-report questionnaires (Ranganath et al., 2008) or subtle behavioral indicators.

Finally, a fruitful avenue for future research may be to make use of experimental approaches to further examine the relationship between negative attitudes and oppression-related representations. By using artificial groups, researchers can create associations linking various groups with negativity and with oppression and then examine whether the novel oppression-related representations suppress the relationship between implicitly measured and explicitly measured attitudes. This approach trades off some degree of real-world applicability to better understand the mechanisms and generalizability of the effect, and thus would complement the current research.

## Conclusion

Although implicit bias remains a widely cited concept in both the scientific literature and public discussion of discrimination, debate remains around the precise nature of these attitudes and their validity in predicting relevant outcomes. In this work, we focused on one potential ambiguity inherent to representations of disadvantaged groups, examining the role of oppression-related representations in implicit attitudes. Building on work demonstrating that representations of oppression relate positively to implicitly measured prejudice but negatively to explicitly measured prejudice, we find that these representations decrease the predictive validity of implicit measures by statistically suppressing the relation between implicit and explicit measures. Decomposing the variance of the standard IAT into variance that is related to oppression and variance that is independent of oppression therefore provides increased predictive validity and, we suggest, a better measure. Thus, although these findings suggest an issue with the current standard implementation of the IAT, they also provide a potential solution that allows the IAT to better capture the representations it aims to measure. Our findings have implications both for implicit measures of prejudice, suggesting that adequate measurement requires researchers to control for contaminating representations, and for debates on the nature of these attitudes, suggesting that dichotomous concepts of valence are not enough to fully understand them. Instead, a fuller picture of one’s semantic associations, including the relation between valenced attitudes and more specific beliefs (Kurdi et al., 2019), is needed to understand what any particular score means. This work represents an initial step in that direction, demonstrating one case in which an implicitly measured attitude (linking Black people with general negativity) and a more specific belief (linking Black people with oppression) are positively correlated but nevertheless have opposite relations with explicitly measured prejudice. More fully mapping out the network of semantic representations underlying attitudes, as well as how these representations interface with more reflective cognitive processes, will be a necessary step in better understanding and reducing prejudice.

## Supplemental Material

sj-docx-1-psp-10.1177_01461672231156029 – Supplemental material for Decoupling the Conflicting Evaluative Meanings in Automatically Activated Race-Based Associations Supplemental material, sj-docx-1-psp-10.1177_01461672231156029 for Decoupling the Conflicting Evaluative Meanings in Automatically Activated Race-Based Associations by Suraiya Allidina, Elizabeth U. Long, Wyle Baoween and William A. Cunningham in Personality and Social Psychology Bulletin

## Notes

1. We use the term “association” to refer to the associative nature of the IAT as a task (i.e., with a single button press used to respond to two different concepts, without assessing any relational information between these concepts). These measured associative responses could arise from multiple types of cognitive representations, including those that are associative and/or propositional.

2. While we focus our discussion on associations of Black people with oppression, the relative nature of concepts like oppression and privilege mean that this framing could easily and equivalently be substituted with associations of White people with privilege, with no loss of meaning.

3. Our preregistration stated that we would first attempt to run our models as structural equation models using four parcels of each of the two critical IATs and two parcels of each of the two prejudice scales, and that, failing satisfactory model fit to create these latent variables, we would instead use linear regression models. However, subsequent reading indicated that recent method articles have demonstrated the unreliability of parcel analysis (Sterba, 2019), and so we instead simply report the linear regressions and mediations. However, results replicate using SEMs (see Supplementary Results), and analysis code to run these models can be found on the OSF page for this project. All other preregistered analyses are reported. We used publicly available stimulus materials (see “Procedure” section), so study materials are not included on the OSF page.

4. Our preregistration is in the form of two parts—the first to document the data splitting and the second to more specifically document the hypotheses and analyses. The first part of the preregistration correctly states that we had already collected an initial sample of 300 participants to form the sample for Study 1. We made an error when reporting the sample sizes again in the second part of the preregistration, where we incorrectly stated that we had already collected 250 rather than 300 participants. The sample size of 300 is the correct number that we first preregistered.

5. Although Black participants on average tend to show low IAT effects, the logic of our suppression analyses should nevertheless apply to their IAT scores. A Black participant who has no anti-Black implicit attitudes could still have a positive score on the IAT due to their associations of Black people with oppression. Thus, the critical suppression analyses are not limited to non-Black participants only.

6. We chose to use 3,000 ms instead of 10,000 ms as our trial exclusion cutoff in our main results because we felt that a latency as high as 10,000 ms suggested that the participant was not paying attention on that trial, rendering it invalid for measuring attitudes.

7. One may wonder whether these effects are unique to the Oppression–Privilege IAT, or whether a measure like the IMS may play a similar role in suppressing the relation between implicit and explicit measures of prejudice. However, a key feature of a suppressor in this context is that it correlates positively with scores on the Black–White IAT, but negatively with scores on explicit measures, leading to a negative indirect effect. The IMS has a negative correlation with Black–White IAT scores, and, therefore, cannot act as a suppressor.

8. Given that the proposed mechanism here involves a relationship among semantic representations, it is unclear how this would play out in measures that have a proposed affective mechanism such as the Affect Misattribution Procedure (Payne et al., 2005).
