# Supplementary material for: Decoupling the Conflicting Evaluative Meanings in Automatically Activated Race-Based Associations
Source: Pers Soc Psychol Bull. 2023 Feb 27;50(7):987–1005. doi: 10.1177/01461672231156029 (PMC11143765; doi:10.1177/01461672231156029)
Supplement: sj-docx-1-psp-10.1177_01461672231156029 – Supplemental material for Decoupling the Conflicting Evaluative Meanings in Automatically Activated Race-Based Associations [file sj-docx-1-psp-10.1177_01461672231156029.docx]

Supplementary Results and Discussion

*Structural equation models*

Our results replicate when fit as structural equation models with the two versions of each IAT (congruent-first and incongruent-first) used as indicators for latent IAT variables. Two indicators were used rather than the planned parcel method suggested by Cunningham et al. (2001) as recent methods papers have demonstrated the unreliability and parameter and fit statistic variance that comes from parcel analysis (Sterba, 2019; Sterba & Rights, 2017). R code for these models is provided on the Open Science Framework entry for this paper. Due to the smaller sample size of the exploratory first study, we report results from only study 2 here. The model had good model fit, SRMR = 0.015, CFI = 0.997, RMSEA = 0.024, 90% CI [0.000, 0.061] and revealed that the Black-White IAT had a positive total effect on explicit attitudes (*b* = 1.083, *SE*=0.249, *z*=4.346, *p* < .001) but a negative indirect effect through the Oppression-Privilege IAT (*b* = -1.664, *SE*=0.563, *z*=-2.955, *p*=.003).

*An arbitrary metric?*

One criticism of the IAT has been that because it is scored on an arbitrary metric, any direct interpretation of scores is difficult. Although one can assign statistical measures of strength (a .8 standard deviation difference between conditions is a “large” effect) to the IAT, whether these metrics translate to psychologically large biases or not cannot be immediately inferred. Given that the measure is response latency based, different people may have different upper bounds - one person’s 300ms difference may simply reflect something different than another person’s 300ms difference. It is therefore difficult to interpret the magnitude of any given score on the IAT, and determining whether a score is “large” or “small” has as of yet been left to more or less arbitrary benchmarks. To address this potential problem, we are able here to calibrate IAT scores with a metric that should conceptually be one’s attitudinal upper bound score - the degree to which people associate positive and negative words with positive and negative pictures (i.e., their scores on the Positive-Negative IAT).

To remove, at least in part, the problems associated with an arbitrary and potentially varying upper bound on the IAT, we created a calibrated score for each participant by dividing the Black-White IAT score by the Positive-Negative IAT score. Conceptually, this standardizes the IAT to reflect how far away each person scored from their theoretical upper bound (a score of 1 would indicate that associations of Black people compared to White people are as negative as possible, whereas a score of .5 would indicate 50% negativity). On average, scores on the Black-White IAT were 21.5% as large as scores on the Positive-Negative IAT. Interestingly, by calibrating the IAT to an objective upper bound, we found that the magnitude of the IAT scores somewhat parallels the results on the explicit measures of prejudice, where the average scores are 19.6% of the scale’s maximum for Blatant Prejudice and 33.7% of the maximum for Symbolic Racism, suggesting that average magnitudes are similar for the two types of measurement.

These calibrated scores can also be used to address another potential problem with the IAT - the idea that individual differences in cognitive functions like executive control may influence IAT scores (Gawronski, 2019). Previous research has suggested that individuals with better executive control abilities may do better on the IAT (i.e. have scores indicating less prejudice) not because they lack the prejudiced association, but because they are better at inhibiting the prepotent response linking a group with their stereotyped attribute (Conrey et al., 2005; Gawronski, 2019). As these cognitive abilities should similarly affect scores on the Positive-Negative IAT, calibrating scores on the Black-White IAT by this theoretical upper bound should allow us to control for any such individual differences in executive functioning. Using these calibrated scores in place of the raw IAT scores does not increase the relationship between implicit scores and explicit measures (see Supplementary Table 3), supporting the conclusion that the D-score method effectively accounts for these kinds of individual differences (Cai et al., 2004). This calibration to a theoretical upper bound, though still useful for interpreting an otherwise arbitrary metric, is therefore not necessary to account for individual differences.

*Meaning of oppression-related associations*

Although it is tempting to consider the Oppression-Privilege IAT to be a relatively direct measure of automatic egalitarianism, it is possible that, just like with the Black-White IAT, its meaning is not uniform. Just as the Oppression-Privilege IAT suppresses the relationship between the Black-White IAT and explicit measures, the Black-White IAT may also suppress the relationship between the Oppression-Privilege IAT and explicit measures, such that the association between the two becomes stronger when variance shared with the other IAT is accounted for. Indeed, examining Table 3 suggests that the standardized relationship of the Oppression-Privilege IAT and explicit measures after controlling for the Black-White IAT is stronger than the zero-order correlation between the two.

To more formally test this idea, we re-ran the suppression analyses using the Oppression-Privilege IAT as the predictor and the Black-White IAT as the suppressor. Results of this analysis are presented in Supplementary Table 4, and suggest that the Black-White IAT does indeed suppress the relationship between the Oppression-Privilege IAT and explicit measures. This may suggest that for some participants the association of White with privileged and Black with oppressed may reflect or contribute to beliefs that are consistent with racism rather than anti-racism. In other words, a greater awareness of the oppression faced by Black Americans (and the relative privilege enjoyed by White Americans) does not on its own entail a belief that this disparity is unjust or harmful and may in fact entail the opposite in some cases, due to halo effects (Nisbett & Wilson, 1977), system justifying beliefs (Jost et al., 2004), or a ‘might=right’ mentality. More research into the broader network of associations underpinning prejudice and social categories will be needed to fully decouple these effects.

References

Cai, H., Sriram, N., Greenwald, A. G., & McFarland, S. G. (2004). The Implicit Association Test’s d measure can minimize a cognitive skill confound: Comment on McFarland and Crouch (2002). *Social Cognition*, *22*(6), 673–684. https://doi.org/10.1521/soco.22.6.673.54821

Conrey, F. R., Gawronski, B., Sherman, J. W., Hugenberg, K., & Groom, C. J. (2005). Separating multiple processes in implicit social cognition: The quad model of implicit task performance. *Journal of Personality and Social Psychology*, *89*(4), 469–487. https://doi.org/10.1037/0022-3514.89.4.469

Cunningham, W. A., Preacher, K. J., & Banaji, M. R. (2001). Implicit attitude measures: Consistency , stability, and convergent validity. *Psychological Science*, *12*(2), 163–170.

Gawronski, B. (2019). Six lessons for a cogent science of implicit bias and its criticism. *Perspectives on Psychological Science*, *14*(4), 574–595. https://doi.org/10.1177/1745691619826015

Jost, J. T., Banaji, M. R., & Nosek, B. A. (2004). A decade of system justification theory: Accumulated evidence of conscious and unconscious bolstering of the status quo. *Political Psychology*, *25*(6), 881–919. https://doi.org/10.1111/j.1467-9221.2004.00402.x

Nisbett, R. E., & Wilson, T. D. W. (1977). The halo effect: Evidence for unconscious alteration of judgments. *Journal of Personality and Social Psychology*, *35*(4), 250–256. https://doi.org/10.1016/0006-8993(93)91773-L

Sterba, S. K. (2019). Problems with rationales for parceling that fail to consider parcel-allocation variability. *Multivariate Behavioral Research*, *54*(2), 264–287. https://doi.org/10.1080/00273171.2018.1522497

Sterba, S. K., & Rights, J. D. (2017). Effects of parceling on model selection: Parcel-allocation variability in model ranking. *Psychological Methods*, *22*(1), 47–68. https://doi.org/10.1037/met0000067

Supplementary Table 1

*Study 1: Means, standard deviations, and correlations with confidence intervals (White Participants)*

| Variable | *Mean*  *(SD)* | 1 | 2 | 3 | 4 | *Congruent latency* | *Incongruent latency* |
| --- | --- | --- | --- | --- | --- | --- | --- |
|  |  |  |  |  |  |  |  |
| 1. Black-White IAT | 0.30  (0.36) |  |  |  |  | 871.32 | 957.65 |
|  |  |  |  |  |  |  |  |
| 2. Oppression-Privilege IAT | 0.24  (0.36) | .52** |  |  |  | 904.37 | 980.17 |
|  |  | [.41, .62] |  |  |  |  |  |
|  |  |  |  |  |  |  |  |
| 3. Positive-Negative IAT | 1.36  (0.51) | .34** | .28** |  |  | 915.61 | 1390.03 |
|  |  | [.22, .46] | [.14, .40] |  |  |  |  |
|  |  |  |  |  |  |  |  |
| 4. Symbolic Racism | 1.05  (0.73) | .25** | -.03 | .03 |  |  |  |
|  |  | [.11, .38] | [-.17, .11] | [-.11, .17] |  |  |  |
|  |  |  |  |  |  |  |  |
| 5. Blatant Prejudice | 1.22  (0.99) | .21** | -.02 | -.04 | .71** |  |  |
|  |  | [.07, .34] | [-.16, .12] | [-.18, .10] | [.63, .77] |  |  |
|  |  |  |  |  |  |  |  |

*Note.* *SD* is used to represent the standard deviation. Values in square brackets indicate the 95% confidence interval for each correlation. For each IAT, we also indicate the mean latencies in milliseconds for the congruent conditions (i.e. Black/bad, White/good for the Black-White IAT; Black/oppressed, White/privileged for the Oppression-Privilege IAT; and positive picture/positive word, negative picture/negative word for the Positive-Negative IAT) and the incongruent conditions (which correspond to the opposite pairings). * indicates *p* < .05. ** indicates *p* < .01.

Supplementary Table 2

*Study 2: Means, standard deviations, and correlations with confidence intervals (White participants only)*

| Variable | *Mean*  *(SD)* | 1 | 2 | 3 | 4 | 5 | 6 | *Congruent*  *latency* | *Incongruent latency* |
| --- | --- | --- | --- | --- | --- | --- | --- | --- | --- |
|  |  |  |  |  |  |  |  |  |  |
| 1. Black-White IAT | 0.35  (0.35) |  |  |  |  |  |  | 847.95 | 949.83 |
|  |  |  |  |  |  |  |  |  |  |
| 2. Oppression-Privilege IAT | 0.30  (0.35) | .27** |  |  |  |  |  | 877.93 | 966.95 |
|  |  | [.18, .35] |  |  |  |  |  |  |  |
|  |  |  |  |  |  |  |  |  |  |
| 3. Positive-Negative IAT | 1.42  (0.48) | .18** | .27** |  |  |  |  | 893.06 | 1376.72 |
|  |  | [.10, .27] | [.19, .35] |  |  |  |  |  |  |
|  |  |  |  |  |  |  |  |  |  |
| 4. Symbolic Racism | 1.06  (0.74) | .16** | -.19** | -.01 |  |  |  |  |  |
|  |  | [.07, .24] | [-.27, -.10] | [-.10, .08] |  |  |  |  |  |
|  |  |  |  |  |  |  |  |  |  |
| 5. Blatant Prejudice | 1.18  (0.98) | .21** | -.14** | -.01 | .70** |  |  |  |  |
|  |  | [.12, .29] | [-.23, -.06] | [-.10, .07] | [.65, .74] |  |  |  |  |
|  |  |  |  |  |  |  |  |  |  |
| 6. IMS | 4.80  (1.27) | -.14** | .16** | .05 | -.53** | -.68** |  |  |  |
|  |  | [-.22, -.05] | [.07, .24] | [-.04, .13] | [-.59, -.46] | [-.72, -.63] |  |  |  |
|  |  |  |  |  |  |  |  |  |  |
| 7. EMS | 2.23  (1.53) | .15** | .03 | .03 | .25** | .37** | -.27** |  |  |
|  |  | [.06, .23] | [-.05, .12] | [-.06, .12] | [.17, .33] | [.29, .44] | [-.35, -.18] |  |  |
|  |  |  |  |  |  |  |  |  |  |

*Note.* *SD* is used to represent the standard deviation. Values in square brackets indicate the 95% confidence interval for each correlation. For each IAT, we also indicate the mean latencies in milliseconds for the congruent conditions (i.e. Black/bad, White/good for the Black-White IAT; Black/oppressed, White/privileged for the Oppression-Privilege IAT; and positive picture/positive word, negative picture/negative word for the Positive-Negative IAT) and the incongruent conditions (which correspond to the opposite pairings). * indicates *p* < .05. ** indicates *p* < .01.

Supplementary Table 3

*Mediation/Suppression results using explicit prejudice scales as the criterion with IAT scores adjusted by maximum possible score*

|  | Indirect Effect | Direct Effect | Total Effect | Prop Mediated |
| --- | --- | --- | --- | --- |
| Study1 |  |  |  |  |
| Symbolic Racism | -0.141 [-0.302, 0.011] | 0.675 [0.375, 0.994]*** | 0.534 [0.25, 0.813]*** | -0.254 [-0.758, 0.018] |
| Blatant Prejudice | -0.147 [-0.365, 0.053] | 0.794 [0.373, 1.23]*** | 0.647 [0.271, 1.013]*** | -0.22 [-0.75, 0.094] |
|  |  |  |  |  |
| Study2 |  |  |  |  |
| Symbolic Racism | -0.151 [-0.22, -0.09]*** | 0.548 [0.362, 0.744]*** | 0.397 [0.218, 0.589]*** | -0.379 [-0.806, -0.191]*** |
| Blatant Prejudice | -0.181 [-0.28, -0.1]*** | 0.783 [0.533, 1.042]*** | 0.602 [0.363, 0.85]*** | -0.296 [-0.599, -0.153]*** |
| IMS | 0.193 [0.092, 0.308]*** | -0.797 [-1.129, -0.479]*** | -0.604 [-0.929, -0.279]*** | -0.313 [-0.817, -0.13]*** |
| EMS | 0.001 [-0.113, 0.121] | 0.645 [0.242, 1.043]** | 0.647 [0.278, 1.022]*** | 0.004 [-0.218, 0.239] |

*Note.* Scores on the Race IAT and Oppression IAT were divided by scores on the Positive-Negative IAT to account for individual variation in task ability. The Indirect Effect represents the effect of the Black-White IAT on explicit prejudice through the Oppression-Privilege IAT. The Direct Effect represents the unique effect of the Black-White IAT on explicit prejudice after removing variance associated with the Oppression-Privilege IAT. The Total Effect represents the overall effect of the Black-White IAT on explicit prejudice including the direct effect and the indirect effect through the Oppression-Privilege IAT. Prop Mediated describes the proportion of the Total Effect that goes through the mediator. A negative Prop Mediated represents a suppressor effect.

* indicates *p* < .05. ** indicates *p* < .01. *** indicates *p* < .001.

Supplementary Table 4

*Mediation/Suppression results using explicit prejudice scales as the criterion, Oppression-Privilege IAT scores as the predictor, and Black-White IAT scores as the suppressor.*

|  |  | Indirect Effect | Direct Effect | Total Effect | Prop Mediated |
| --- | --- | --- | --- | --- | --- |
| Study1 |  |  |  |  |  |
| Symbolic Racism |  | 0.357 [0.204, 0.543]*** | -0.307 [-0.579, -0.027]* | 0.051 [-0.202, 0.302] | 1.921 [-47.236, 41.015] |
| Blatant Prejudice |  | 0.417 [0.219, 0.655]*** | -0.38 [-0.735, -0.016]* | 0.036 [-0.292, 0.362] | 1.542 [-33.073, 41.107] |
|  |  |  |  |  |  |
| Study2 |  |  |  |  |  |
| Symbolic Racism |  | 0.152 [0.094, 0.223]*** | -0.488 [-0.647, -0.323]*** | -0.336 [-0.498, -0.173]*** | -0.446 [-0.998, -0.226]*** |
| Blatant Prejudice |  | 0.199 [0.123, 0.294]*** | -0.573 [-0.786, -0.354]*** | -0.374 [-0.589, -0.156]*** | -0.524 [-1.379, -0.254]*** |
| IMS |  | -0.198 [-0.302, -0.109]*** | 0.6 [0.32, 0.889]*** | 0.402 [0.134, 0.686]** | -0.485 [-1.671, -0.21]** |
| EMS |  | 0.168 [0.064, 0.296]*** | -0.015 [-0.355, 0.337] | 0.153 [-0.177, 0.495] | 0.694 [-8.755, 11.386] |

*Note.* The Indirect Effect represents the effect of the Oppression-Privilege IAT on explicit prejudice through the Black-White IAT. The Direct Effect represents the unique effect of the Oppression-Privilege IAT on explicit prejudice after removing variance associated with the Black-White IAT. The Total Effect represents the overall effect of the Oppression-Privilege IAT on explicit prejudice including the direct effect and the indirect effect through the Black-White IAT. Prop Mediated describes the proportion of the Total Effect that goes through the mediator. A negative Prop Mediated represents a suppressor effect.

* indicates *p* < .05. ** indicates *p* < .01. *** indicates *p* < .001.
